# Fit and Proper Requirements in the EU Banking Sector. A Step Further

**DOI:** 10.1007/s40804-022-00244-4

**Published:** 2022-04-19

**Authors:** Matteo Arrigoni, Mattia Rivolti

**Affiliations:** 1 Assistant Professor of Corporate Law, Università Cattolica del Sacro Cuore, Milan, Italy; 2 Faculty of Banking, Finance and Insurance Sciences, Università Cattolica del Sacro Cuore, Milan, Italy; 3 Banking Supervisor, European Central Bank (ECB), Frankfurt am Main, Germany

**Keywords:** Fit and proper requirements, CRD IV, Management body members, Key function holders, Chairs of the management body’s special committees, Super experts

## Abstract

Prudential regulation has become increasingly important over time. In such a context, strict suitability requirements for members of the management body and key function holders are considered an adequate tool to safeguard the sound and prudent management of banks. In the years following the Great Financial Crisis in 2008–2009 and the Eurozone sovereign debt crisis in 2010–2012, the regulatory and supervisory approach to banks’ corporate governance has seen a significant evolution. The centralisation of banking supervision at the Eurozone level was complemented by the application of a single rulebook throughout the European Union which stated, in compliance with the Capital Requirements Directive, higher suitability requirements for being appointed as a member of a management body or as a key function holder at a bank. Furthermore, the choice to identify fit and proper requirements through standards attributes significant power to the decision maker, thus allowing greater public control over private choices, and is justified by the need to prevent negative externalities. This approach is open to debate. Against the background of the pursuit of public interest, the lawmaker confers discretionary power on the supervisory authorities (the European Central Bank and the national competent authorities), and sets restrictions on the job market for board member candidates. This paper is a contribution enriching the ongoing discussion about the existing regulatory and policy regime, with a special focus on the specific functions performed by the management body members and particularly by the chairs of the management body’s special committees*.*

## Introduction

Although mainly composed of private companies, the banking sector is characterised by a significant public interest.^1^ The traditional approach identified *ex post* measures as the most balanced strategy for meeting the public interest, while at the same time ensuring maximum freedom of enterprise.^2^ However, *ex post* strategies show some limits, i.e., they do not completely avoid the spreading of negative externalities, such as the contagion risk in the banking sector, and entail high implementation costs.^3^ Hence the rationale for a prudential regulatory strategy, composed of several tools to safeguard the sound and prudent management of banks.

In this perspective, the prudential regulation of banks’ capital takes on a central role; prudential capital has been set as a burden for risk-taking and allows individual banking risks to be managed. As required by the European lawmaker, credit institutions must ‘have internal capital that, having regard to the risks to which they are or may be exposed, is adequate in quantity, quality and distribution’.^4^ Similarly, poor and inadequate risk management practices showed weaknesses in the governance of credit institutions,^5^ and this prompted new developments in the field of corporate governance. In fact, in addition to solving the typical agency problems within a company, corporate governance is also key to managing risks deriving from the banking business.^6^ Hence, the regulatory provision stipulates that banks

shall have robust governance arrangements, … effective processes to identify, manage, monitor and report the risks they are or might be exposed to, adequate internal control mechanisms, including sound administration and accounting procedures, and remuneration policies and practices that are consistent with and promote sound and effective risk management.^7^

In such a context, the suitability requirements for members of management bodies and key function holders act as the pillar of banking corporate governance. Therefore, the supervision of the suitability of individuals ultimately responsible for the bank’s business and its related risk-taking appears to be an adequate tool to safeguard the sound and prudent management of credit institutions.

In the years following the Great Financial Crisis and the Eurozone sovereign debt crisis, this approach has been subject to significant developments. The establishment of the Banking Union centralised the supervision of the Eurozone banks within the European Central Bank (hereinafter ‘ECB’).^8^ The two pillars of the Banking Union rest on the foundation of the single rulebook, which applies to all EU countries (e.g., stricter suitability requirements as set forth in the CRD IV for candidates for management bodies or key function holder positions of a bank). Furthermore, the use of standards for determining fit and proper requirements confers significant power on the decision maker^9^ and is justified by the need to prevent negative externalities from non-prudent risk management of the banking business, in particular during crisis periods. This ensures greater public control over private choices as well as flexibility in the selection process for the shareholders.

The approach defined by the CRD IV is open to debate. Although justified by the pursuit of public interest, it may provide the supervisory authority^10^ with excessive discretion power and carry the risk of arbitrariness and unjustified constriction of the freedom to conduct a business. An excessive pervasiveness of the requirements for performing the role of management body member also entails high access costs, thus narrowing its job market. In addition to the risk of not finding adequate candidates, this pervasiveness also creates new market failures, such as the emergence of new potential areas of power managed by head hunting companies whose interest may not coincide with the collective interest, producing candidates who, while compliant with the framework, are unable to implement sound and prudent management practices. Finally, the application of the requirements to any member, regardless of the specific position held, entails a cost not balanced by the benefits, creating a disproportionate solution with respect to the problem.

Thus, there is room for a step further. The current regulatory framework could be improved by identifying individual criteria also in combination with the specific role performed, and with a special focus on the responsibilities held by management body members and particularly by chairs of the management body’s special committees. In this regard, without prejudice to the necessary discretion provided to the supervisory authority to duly perform its supervisory tasks, a new framework could better balance the private interest of adequate control and the public interest of an efficient solution to manage the risks in the market.

The article is organised as follows. After a description of the criticalities of a fit and proper requirements framework in Sect. 2, Sect. 3 illustrates in detail the provisions of the CRD IV and its implementing acts. A critical evaluation of the existing regime is offered in Sect. 4. Section 5 illustrates the possible solutions offered by the current legal system to address the problems highlighted. From a policy perspective, Sect. 6 puts forward an alternative view. Section 7 concludes.

## The Problem of Guaranteeing Fundamental Rights

The suitability requirements for members of the management body and key function holders are considered an adequate safeguard to ensure a sound and prudent management of banking activity.^11^ Therefore, the European lawmaker states that

the shareholders or members of an institution should consider whether the candidates [for being members of the management body] have the knowledge, qualifications and skills necessary to safeguard proper and prudent management of the institution.^12^

Although justified by the pursuit of the public interest to preserve the stability of and confidence in the banking system, as a matter of fact, the law makes the performance of the banking business subject to meeting certain requirements, which therefore involves a constriction of the freedom to conduct a business. This could raise some criticism regarding the compliance with constitutional provisions. Within the European Union, this freedom is protected by Art. 16 of the Charter of Fundamental Rights of the European Union (hereinafter ‘CFREU’) and by the European Convention for the Protection of Human Rights and Fundamental Freedoms (hereinafter ‘ECHR’). In this regard, ‘the Union shall accede’ to the ECHR,^13^ while the CFREU, starting from the Lisbon Treaty, ‘shall have the same legal value as the Treaties’.^14^ Moreover, ‘fundamental rights, as guaranteed by the [ECHR] and as they result from the constitutional traditions common to the Member States, shall constitute general principles of the Union’s law’.^15^

Still, the freedom to conduct a business is not absolute. In fact, the protection of a specific constitutional freedom could lead to prejudice to another freedom or a general interest. Hence the legitimacy of a constrained freedom, provided that an adequate balance between potentially conflicting interests is guaranteed. From this perspective,

it likewise seems legitimate that these rights should, if necessary, be subject to certain limits justified by the overall objectives pursued by the Community, on condition that the substance of these rights is left untouched.^16^

All the laws about the suitability requirements for members of management bodies and for key function holders therefore are along these lines aimed at safeguarding the general interest, without disproportionately constraining related private interests. In a nutshell, potential criticalities may affect the reconciliation between the rule of law principle on which the suitability requirements are grounded and the need for framework flexibility and effective oversight to adapt to the specific banking context.

## The EU Fit and Proper Requirements

According to the general framework, throughout the European Union, ‘members of the management body’ have to be of ‘sufficiently good repute and possess sufficient knowledge, skills and experience to perform their duties.^17^ The subsequent provisions specify that all management body members ‘shall commit sufficient time to perform their functions’, have a limited ‘number of directorships’ and ‘act with honesty, integrity and independence of mind’.^18^ With reference to the overall composition of the management body, the board ‘shall reflect an adequately broad range of experiences’ and

possess adequate collective knowledge, skills and experience to be able to understand the institution’s activities, including the main risks. The overall composition of the management body shall reflect an adequately broad range of experience.^19^

For these purposes ‘institutions and their respective nomination committees’ shall ‘engage a broad set of qualities and competences when recruiting members to the management body’.^20^

Moreover, these requirements are specified by the European Banking Authority (EBA) and the European Securities and Markets Authority (ESMA) guidelines,^21^ whose approach is a combination of standards and rules. Therefore, along with the wide use of standards, many provisions ensure discretionary powers for the supervisory authority in assessing the fit and proper requirements for candidates.

For example, in assessing the sufficient time commitment of a proposed board member, among other elements, the following must be considered: the member’s geographical presence and the travel time required for the role; the number of meetings scheduled for the management body; and the necessary induction and training.^22^ Therefore, these provisions allow the supervisory authority to ‘apply a case-by-case approach’.^23^ Or, with reference to the practical and professional experience of management body members, they must have gained such experience from a managerial position over a ‘sufficiently long period’.^24^ Moreover, to assess the members’ reputation, honesty and integrity in addition to convictions for specific offences, the following must be considered: ‘ongoing prosecutions for a criminal offence’; financial and business performance of entities owned or directed by the member, civil lawsuits, and administrative or criminal proceedings.^25^ To assess independence of mind, it has to be judged if all members of the management body have the necessary behavioural skills, including: ‘courage, conviction and strength to effectively assess and challenge the proposed decisions of other members of the management body’; the ability ‘to ask questions to the members of the management body in its management function’; and the ability ‘to resist “group-think”’.^26^ Finally, the assessment of competences is qualitative, as there are no presumptions based on specific examinations.

The legislation described above is in force across the European Union and therefore the Eurozone as well. In this regard ‘the ECB should be able to exercise supervisory tasks in relation to all credit institutions authorised in, and branches established in, participating Member States’.^27^ Therefore, the ECB oversees banks’ compliance with all relevant Union laws (including national legislation transposing EU directives) which impose requirements on credit institutions, such as ‘the fit and proper requirements for the persons responsible for the management of credit institutions’.^28^ In this context, the ECB published two Decisions aimed at regulating its assessment process^29^ and a Guide for harmonising the criteria underlying its activity.^30^ The Guide is not legally binding and does not replace EU laws or national transposition laws. However, it retains some relevance because it limits the ECB’s discretion in two ways.^31^ Firstly, it recalls the purpose of the FAP requirements according to which the ECB plays a gatekeeper role in preventing individuals who would pose a risk to the proper functioning of the management body from joining the board or from continuing in their role in the light of new facts impairing their fitness and propriety; complementarily, the ECB supervises credit institutions’ compliance with the requirements regarding robust governance arrangements.^32^ Secondly, the Guide specifies some standards of the Joint ESMA and EBA (2021); for example, experience is assessed against guiding presumptions of sufficient experience based on thresholds (e.g., the presumption of adequate experience for the CEO is ten years of recent practical experience in areas related to banking or financial services, including a significant proportion of senior level managerial positions).^33^ Nonetheless, a combined analysis of the two documents (Joint ESMA and EBA 2021 and ECB 2021) shows that the supervisory authority is still empowered to exercise supervisory judgement. Consider, among many examples, the evaluation of ongoing prosecutions for a criminal offence, where ‘the very fact that an individual is being prosecuted is relevant to propriety. In the assessment, all existing information regarding the propriety of the appointee together with the stage of the proceedings and the evidential weight of the alleged wrongdoing should be assessed’.^34^ In such cases, the verification of correctness is not automatically anchored to a specific stage of the judicial authority’s restrictions^35^ and the ECB could find itself in the situation of taking into consideration charges without the certainty of their confirmation in the following or in a subsequent court ruling.^36^

## A Critical Evaluation

Although founded on scientific studies demonstrating how the skills and experience of management affect the sound or poor performance of a credit institution,^37^ the lessons learnt from the past years of application of the FAP regime as well as the continuous evolution of the banking sector offer some additional points for reflection for the EU lawmaker.^38^

First, potentially excessive discretion left to the supervisory authority may entail the risk of arbitrariness and errors, also considering that ‘the prudential and resolution authorities are staffed by humans—and we humans are prone to make mistakes and are guided by assumptions and biases, cognitive and social.’^39^ This entails both a further reputational risk for the supervisory authority due to potentially controversial decisions and a possible infringement of the rights of the addressees of the decisions or, better, an unjustified constriction of their freedom to conduct a business.^40^

Second, the targeted specialisation of the requirements may imply challenges in selecting board members with proper technical skills (e.g., NPL management and recovery skills, digitalisation, IT, and climate-related and environmental risk knowledge) due to a limited number of competent and experienced candidates, especially for banks with complex business structures (e.g., global systemically important banks—G-SIBs) as well as for banks operating in smaller countries subject to recent financial crises (e.g., banks conducting business in Portugal, Greece and Cyprus); consequently, targeted specialisation may place great importance on head hunting companies advising banks about candidates for the role of management body members. In fact, head hunting companies may be helping to allocate highly specialised and sometimes scarce resources to targeted banks, in particular in markets that are not particularly competitive (e.g., when there are remuneration constraints for crisis banks under restructuring plans, but which are searching for very skilled board members). In this perspective, the advice of head hunters may turn into a sort of ‘certification’ regarding the role of board members or key function holders, with a clear risk of violating the democratic principle according to which the powers to limit individual freedoms must be attributed to subjects who, directly or indirectly, are representatives of the popular will under specific constraints.

Finally, without prejudice to the establishment of a minimum level of provisions, the application of the FAP requirements should be targeted as much as possible at the position held by each member to avoid costs that are not fully balanced by benefits, creating a disproportionate solution with respect to the problem. In fact, each member is supposed to play specific roles as defined by the bank’s internal governance model and rely on the diversity of their backgrounds and contributions to the sound and prudent management of credit institutions. Indeed, the board could carry out different functions, being either a ‘monitoring board’ charged with a controlling role or a ‘managing board’ tasked with an active role in management.^41^ In doing so, banks confer a specific mandate on executives and non-executives, the latter being appointed as members of the management body’s special committees. In this regard, the legal framework does not provide for different or enhanced technical and soft skills for the key role of special committees’ chairs vis-à-vis those of the other members.^42^ Conversely, the lawmaker is more focused on the contribution made within the special committees by formal independent board members whose qualification is centred on the nature of the relationship with the bank (i.e., free of conflicts of interest) and is different across EU jurisdictions.^43^

## Fundamental Rights Protection and the Principle of Proportionality as Solutions to the Problems Described

The protection of fundamental rights can offer a solution to the problem of potentially excessive discretion provided to the supervisory authority.

In fact, in cases of serious interference with fundamental rights the Court has repeatedly affirmed the need for a more stringent scrutiny of decisions, as well as reduction of the regulatory margin of discretion. In other words, authorities’ decisions pursue a public interest that must be balanced with the private interest of the protection of fundamental rights.^44^ In this regard, in assessing whether there has been a violation of a fundamental right or freedom, the analysis must be brought back to compliance with the principle of proportionality.^45^

In this context, the principle of proportionality should not be understood as a general principle that binds all institutions of the European Union, based on which the European Union Court of Justice checks that an act is appropriate to achieve its objectives and does not exceed the limits imposed.^46^ Rather, when the interests involved are private and public, the proportionality principle involves assessing whether, to pursue the public interest, an excessive burden has been imposed on individuals or not, since if an excessive burden has been imposed the right balance required will not be reached.^47^ More specifically, ‘in considering whether the interference has imposed an excessive individual burden’, the Court will take into account ‘the particular context in which the question arises’, drawing from the peculiarities of the concrete case the arguments confirming or disproving the legitimacy of the decision taken by the supervisory authorities.^48^

As regards the subject of this paper, the principle of proportionality is applied, in different ways, to the individual requirements necessary to manage a bank. For example, the principle of proportionality is not relevant for the reputation requirement because ‘a person has either a good or a bad reputation’;^49^ on the other hand, regarding the theoretical knowledge and practical experience requested, the principle of proportionality is applicable, as‘ [t]he more complex these characteristics are, the more experience will be required’.^50^

Regarding the other two critical points raised—the potentially excessive pervasiveness of the requirements and the need for a more detailed approach depending on the role played—the ECB itself stated that ‘the principle of proportionality applies throughout the whole fit and proper process’ and, therefore, ‘the application of the suitability criteria should be commensurate with the size of the entity and the nature, scale and complexity of its activities, as well as the particular role to be filled’. The result could be

a differentiated approach to the assessment procedure or the application of suitability criteria (e.g. in terms of the level or areas of knowledge, skills and experience, or in terms of the time commitment required of members of the management body in its management function and members of the management body in its supervisory function).^51^

Hence, there is the possibility of a graduated approach that avoids disproportionate measures.

However, the solutions offered by the lawmaker show room for improvement, since they lead to the application of the principle of proportionality, which is endowed, by definition, with discretion. In other words, the lawmaker ends up re-proposing an unclear outline, thus not eliminating the potentially excessive discretion that one wanted to avoid. With reference then to the administrative discretion, judicial control is not exhaustive, and only allows control of the reasoning by the supervisory authorities in the case of a ‘manifest error of assessment’. Although justified by the intention of confining scrutiny of independent authorities’ choices to only exceptional hypotheses to maintain efficiency and institutional balance, the preference for jurisdictional control limited by the principle of proportionality may therefore be insufficient, also considering the ‘deferential attitude’ of the Court when decisions with technical discretion are involved.^52^ Thus, a policy solution follows.

## Policy Proposals to Improve the Existing Framework

The importance of offering policy proposals aimed at improving the current regulatory system is confirmed by the consideration that the European financial sector is rapidly evolving.^53^

To reach this aim, it is important to note that a diametrically opposite approach—rules-based vis-à-vis principles-based—is not fully desirable either. First, a law based solely on rules presents the problem of obsolescence. Furthermore, the discretion of standards allows a better adaptation to concrete cases^54^ (for example, this has allowed the Greek national law to adapt the FAP requirements to the specific needs within the national banking sector, e.g., board members with specialised knowledge and experience, as well as enhanced independence criteria following the third bailout^55^). Lastly, the specificity of some requirements—such as a minimum level of skills—is indispensable in a technical market such as banking.

From another point of view, the need to avoid the negative externalities caused by a bank crisis, and the objective of eliminating so-called competition in laxity^56^ suggest an opportunity for harmonisation of criteria at European level^57^ based on best practices achieved. Indeed, the current system is based on the transposition of the CRD IV provisions at the national level, leading to different results depending on the Member State where the bank is located.^58^ Furthermore, a unique system of rules fosters real competition between banks’ board member candidates and allows the supervisory authority to perform its supervisory role with greater efficiency.^59^ However, the loss of regulatory power at the national level has always been a political problem.^60^ Therefore, there is an increased urgency to avoid possible abuses by the supervisory authority. Limiting its discretion not only is functional to a greater protection of the subjects involved, it is also a balanced compromise with the Member States, which might be more prepared to give up part of their sovereignty (i.e., the identification of FAP requirements by national law) in exchange for a greater level playing field applied by the ECB (in the Banking Union) or by the NCAs (outside the Banking Union but within the European Union).

It seems feasible to take a step further in the FAP requirements considering that there is an urgent need to anchor the meaning (and verification) of the requirements and criteria analysed in this paper to mandatory and objectively verifiable circumstances, in order to guide the judgements of supervisory authorities,^61^ and seeing that the available empirical literature on governance failures in the run-up to the financial crisis appears to be rather inconclusive.^62^ Moreover, the tightening of the FAP requirements demonstrates the preference for a preventative approach more oriented towards the protection of stability, which at the same time entails higher access costs, with a consequent reduction in the supply of candidates who intend to perform management roles in banks, therefore decreasing competition in the market. Lastly, if the suitability requirements are considered as an instrument to safeguard the sound and prudent management of banks, then they must be calibrated to the different functions performed by board members.

From this point of view, the FAP requirements could benefit from the following policy proposals.

First, with reference to the requirement of good repute,^63^ different strategies could be used to limit the discretion of the supervisory authority. In fact, as regards the request according to which the terms related to good repute ‘should be defined and explained in a consistent way’,^64^ it is possible to propose a different solution primarily aimed not at further specifying or restricting their legal definition, but rather at increasing the supervisory authority’s accountability through compliance with more precise drivers supporting the FAP assessment. In this perspective, the supervisory authority could indeed consider as relevant ‘all existing information regarding the propriety of the appointee together with the stage of the proceedings and the evidential weight of the alleged wrongdoing’;^65^ however, the requirement of good repute should only be missing when the facts or issues that may be relevant to the assessment of an appointee’s reputation describe a material, precise and concordant picture of conduct that goes against the sound and prudent management of the bank.^66^ Overall, higher accountability achieves the twofold objective of safeguarding the protection of the addressees of the decisions as well as market confidence in the supervisory authority.

Furthermore, there is the provision of more discretionary norms (and therefore the use of standards) for the verification of knowledge, skills and experience, since the greater flexibility of this approach allows the norms to evolve and adapt to changing circumstances (e.g., NPL management, derivatives, fintech applications, such as IT risk and digitalisation, climate-related and environmental risks).

However, even in the latter case it is possible to limit the discretion of the supervisory authority according to the following recommendation.

First, the role held by the chairs of the management body’s special committees (e.g., risk, internal audit, compliance & ethics, remuneration, and nomination committees) in leading and facilitating the decision-making process of the overall management board should be recognised. Without prejudice to the ultimate responsibility of the full management body, the chairs of special committees hold a crucial role in informing and guiding the full management body in its final decision or in its ratification of delegated decisions suggested by the special committees. Thus, the complexity of the decision-making process as well as the highly specific technical matters require specialist chairs of special committees (i.e., ‘super experts’) equipped with both soft skills (e.g., steering skills), partially like those needed for the chairing of the full management body, and specific technical skills (e.g., for NPL management, digitalisation, or climate-related and environmental risks).^67^

From this point of view, it seems reasonable to distinguish the knowledge, skills and experience requirement in relation to the role played by a board member of a bank. For example, non-executive directors have to oversee and control the management of banks without performing management actions themselves—thus they should have the necessary skills to follow their ‘nose in/fingers out approach’, such as ‘knowledge in the areas of risk appetite-setting and management, and in auditing and compliance’.^68^ On the other hand, the chairs of special committees play a key role in the oversight of how related activities are managed. In this regard, the current framework identifies, as additional qualifications for them, the requirement of formal independence, by focusing on the mitigation of potential conflicts of interest, in which regard the national legislation foresees formal independence. However, chairs’ effective and successful performance in steering the special committees’ decision-making already requires additional professional qualifications in terms of both soft and hard skills. Therefore, the imposition of additional requirements for the chairs of special committees would adapt the framework to the best practices implemented by banks.

Furthermore, this policy proposal would also guide the bank’s recruitment procedures in selecting super experts with the aim of deeply relying on their technical and managerial competences via their appointment as chairs of special committees. Against the background of a limited roster of suitable candidates, the super experts could be incentivised to accept roles which imply a real assumption of responsibilities based on specific competences. This approach would also be helpful for banks subject to restructuring and salary caps for board members, where the need for special skills (e.g., the need for NPL management and recovery skills in the Greek, Italian or Portuguese banking sectors) does not easily match with the availability of local candidates, thus urging the selection of foreign board members who developed such skills in different banking sectors (e.g., who acquired NPL management and recovery skills in the Irish or Spanish banking sectors).

In fact, the need for super experts could imply a trade-off between specialised skills (technical and soft) and knowledge of the banking sector in cases where banks would opt for selecting foreign board members. In such situations, on top of dedicated training sessions, the chairs of special committees could be effectively supported by local board members with a diverse yet complementary professional background, i.e., adequate knowledge of the real economy and business dynamics in targeted geographic areas (e.g., local bankers or entrepreneurs). Therefore, the diversity of board members appears to provide added value.

As a result, the management board’s decision-making would be able to count on a sound mix of qualifications effectively allocated to the key roles within the full board and its special committees, with beneficial effects on both the supervisory authority and the bank. In fact, on the one hand, the supervisory authority would identify competent and accountable (within their organisational structure) reference persons at management body level, i.e., the super expert chairs; on the other hand, the framework would be adapted to the actual qualifications requested by banks for the roles of the chairs, thus acknowledging and prioritising banks’ efforts in selecting super experts.

Two additional suggestions regarding the approval process would make the process even more efficient. First, the approval of a board member should be formally granted by the supervisory authority only *ex ante*, i.e., always before the actual appointment by the bank. In the current system, in fact, each Member State can decide whether the assessments of the suitability of members of the management body should be made before or after the appointment.^69^ The underlying reason is very straightforward: the costs of such an approach do not seem higher than the benefits. As emerged from the discussion related to this topic in the Joint ESMA and EBA (2017),^70^ ‘an *ex-ante* assessment would provide more certainty to the institution and also avoid reputational risk for the competent authorities’.^71^ On the other hand, ‘*ex-ante* assessment by competent authorities could lead to higher costs for institutions due to the longer recruitments process for institutions’; moreover, in ‘Member States where the number of institutions is high … such an approach would be costly and burdensome’; lastly, ‘competent authorities that perform *ex-post* assessments would need to change their procedures and changes to national laws might be needed’.^72^ In this regard, the issue of too lengthy assessments that ‘would reduce the attractiveness of the positions, leaving key positions unfilled and, at the same time, [that] would hinder changes of control situations where management members are often replaced’^73^ could be solved as follows: the FAP process could include a time limit for the assessment by the supervisory authority beyond which the candidate’s suitability is presumed. Second, more discretionary rules could be provided for when, during an ongoing audit, the supervisory authority detects issues in the institution (indeed, the increase in risk could justify greater pervasiveness of control). Moreover, this last observation requires accurate identification of the events in which the supervisory authority can intervene (so-called trigger events) and could also raise a potential reputational risk for the interested party and the authority; the former would face damage to its image because the decision is made public, while the latter would have to explain why it had given authorisation for the appointment of the person in question at an earlier stage.

## Conclusions

Regulating the suitability constraint of management bodies’ members and key function holders is not a simple task. A law that is too loose may not sufficiently protect the public interest in the sound and prudent management of banks. On the other hand, a law that is too pervasive could cause excessive damage to the freedom to conduct a business. To stay within these limits, the European lawmaker has adopted an approach that is not too excessive on the book, while at the same time entrusting supervisory authorities with the task of pursuing the public interest, using many standards. However, this approach has room for improvements, leveraging on the lessons learnt from the past years of application of the FAP regime as well as from a continuously evolving banking industry. Therefore, a step further could be taken with a balanced solution that develops the current standards, to address the new challenges posed by the banking sector (i.e., new banking businesses and corresponding technologies, Covid-19 pandemic crisis) in full compliance with the level playing field across the European single market. By distinguishing the individual criteria and considering the functions that board members perform, this article is an attempt to improve the current regime, with a special focus on the role played by the management body members and particularly by the chairs of the management body’s special committees.

## Footnotes

1 The need for specific regulation for the banking sector is grounded on multiple reasons. Firstly, in combination with other financial intermediaries, banks play a pivotal role in sustaining the real economy, including deposit-taking, loan-granting and payment services; therefore, the performance of these activities implies a public interest. Secondly, the weaknesses within the banking sector could negatively impact the economic sector, as happened with the credit crunch during the Great Financial Crisis in 2008–2009; hence the need to prevent negative externalities. Furthermore, systemic risks are potentially embedded in the banking business itself: due to the potential maturity mismatch in their balance sheets (i.e., short-term liabilities v. long-term assets), banks may be exposed to a so-called ‘bank run’ following adverse events. Finally, their inherent interconnection exposes every bank to the risk of shocks triggered by other banks.

2 For example, the approach behind the role of ‘lenders of last resort’ given to central banks, a role firstly identified by Thornton (1802) and elaborated later by Bagehot (1873). On this point, see Laidler (2003), p 61, and Humphrey (2010), p 333; more recently, Tucker (2014); for a complete comparison, Bordo (1990).

3 In this regard, ‘ex-post’ public bailouts were one of the root causes of the Eurozone sovereign debt crisis.

4 Recital no. 43 of Directive 2013/36/EU, the so-called Capital Requirements Directive IV, hereinafter ‘CRD IV’.

5 Financial Stability Board (2013); Senior Supervisors Group (2009). In the words of the ECB: ‘The financial crisis identified significant weaknesses in the functioning and composition of banks’ management bodies. Amongst other factors, these led to shortcomings in risk management, insufficient implementation of sound corporate governance and a lack of effective oversight of management’, ECB (2018a). Additional references can be found in Busch et al. (2019), p 7 f.

6 The transformation of banking governance into banking risk governance, following an approach deemed to ensure stability in the financial system through the sound and prudent management of individual institutions—focusing on the specific characteristics of the banking activity and the potentially arising systemic risks from not very prudent management—is underlined by Frigeni (2016), p 23; see also Hopt (2013); at the institutional level, Organisation for Economic Co-operation and Development (OECD) (2010) and European Commission (2010). Hence the role of the ECB as ‘gatekeeper [that ensures] that banks have robust governance arrangements and suitable board members’, ECB (2018a).

7 Art. 74, para. 1, CRD IV; see also Art. 88 CRD IV.

8 The first pillar of the Banking Union is the so-called Single Supervisory Mechanism (‘SSM’) under which the ECB is the central prudential supervisor of credit institutions in the euro area and in non-euro area countries that choose to join the SSM. The SSM was established by Council Regulation (EU) No. 1024/2013 of 15 October 2013 conferring specific tasks on the European Central Bank concerning policies relating to the prudential supervision of credit institutions (hereinafter ‘SSM Regulation’). On the Banking Union framework see, *ex multis*, Busch and Ferrarini (2020).

9 The cost-benefit analysis and an in-depth description of the characteristics of standards as opposed to rules are a classic in the international literature: see, for all, Kaplow (1992); Sullivan (1992); and lastly, Sunstein (1995). More recently, see Schauer (2004) and Korobkin (2000).

10 Within the SSM, the supervisory authorities are the ECB, which directly supervises the largest banks, and the National Competent Authorities (‘NCAs’), which monitor the remaining banks. This paper is mainly focused on SSM banks and thus investigates the approach taken by SSM supervisory authorities.

11 In this regard, more broadly ‘the first line of defence against financial crises rests with financial institutions themselves, which are responsible for their own safety and soundness’, ECB (2007), p 73.

12 Recital no. 59, CRD IV.

13 Art. 6, para. 2, Treaty on European Union, hereinafter ‘TEU’.

14 Art. 6, para. 1, TEU.

15 Art. 6, para. 3, TEU. See also Lautenschläger, p 28, stating that ‘decisions taken by supervisors may have a considerable impact on the fundamental freedoms and rights of banks, investors and depositors’; see also, more generally, Lamandini (2016), p 20. Moreover, the Court of Justice immediately upheld its jurisdiction on this matter, even before the assimilation of the CFREU with the treaties: see ECJ, 11 January 1977, *J Nold Kohlen und Baustoffgrosshandlung v Ruhrkohle Aktiengesellschaft*, C-4/73, ECLI:EU:C:1974:51, and ECJ, 12 November 1969, *Erich Stauder v Ulm-Sozialamt*, C-29/69, ECLI:EU:C:1969:57.

16 Quote from ECJ, 14 May 1974, *J. Nold, Kohlen- und Baustoffgroßhandlung v Commission of the European Communities*, C-4/73, ECLI:EU:C:1974:51, according to which ‘if rights of ownership are protected by the constitutional laws of all the Member States and if similar guarantees are given in respect of their right freely to choose and practice their trade or profession, the rights thereby guaranteed, far from constituting unfettered prerogatives, must be viewed in the light of the social function of the property and activities protected thereunder. For this reason, rights of this nature are protected by law subject always to limitations laid down in accordance with the public interest’ (on this issue, see, *ex multis*, Gabriël and Trone (2010), p 388). See also ECJ, 13 July 1989, *Hubert Wachauf v Bundesamt für Ernährung und Forstwirtschaft*, C-5/88, ECLI:EU:C:1989:321; ECJ, 30 July 1996, *Bosphorus Hava Yollari Turizm ve Ticaret AS v Minister for Transport, Energy and Communications and others*, C-84/95, ECLI:EU:C:1996:312. In the same sense, the Italian Constitutional Court has repeatedly affirmed that, on the one hand, the freedom to conduct a business is not absolute and, on the other hand, it is possible to make it subject to restrictions, provided that such freedom is not undermined in the substance: see, Corte Cost., case no. 20/1980; Corte Cost., case no. 137/1971; Corte Cost., case no. 38/1960; Corte Cost., case no. 53/1958; Corte Cost., case no. 103/1957.

17 Art. 91, para. 1, CRD IV.

18 Art. 91, paras. 2, 3 and 8, CRD IV.

19 Art. 91, paras. 1 and 7, CRD IV.

20 Art. 91, para. 10, CRD IV, Suitability requirements have existed for some time: initially set forth in Art. 3 of Directive 77/780/EEC (on the ‘principle of four eyes’, see Capolino (2016), p 68), they were subsequently regulated by Arts. 5 and 11 of Directive 89/646/EEC; their framing into the so-called ‘fit and proper requirements’ dates to a later stage due to the Basel Committee on Banking Supervision (1997) and subject to a gradually higher relevance in the subsequent reviews of the Basel Accords.

21 Joint ESMA and EBA (2021). These Guidelines apply as of 31 December 2021 and repeal the previous Joint ESMA and EBA (2017). For delegation to EBA and ESMA, see, respectively, Art. 91, para. 12, CRD IV, and Art. 9, para. 1, second subparagraph, Directive 2014/65/EU (Markets in Financial Instruments Directive, the so-called ‘MiFID II’).

22 No. 41, Joint ESMA and EBA (2021).

23 With reference to the Joint ESMA and EBA (2017), with the same content as the Joint ESMA and EBA (2021), see ECB (2019a). As regards the time commitment assessment the ECB has adopted both quantitative and qualitative criteria, ECB (2019b), p 3 f.

24 No. 64, Joint ESMA and EBA (2021).

25 Respectively, no. 74, let. a, and no. 76, let. b and d, Joint ESMA and EBA (2021).

26 No. 81, let. a, Joint ESMA and EBA (2021).

27 Recital no. 16, SSM Regulation.

28 Art. 4, para. 1, let. e, SSM Regulation.

29 Decision (EU) 2017/935 of the European Central Bank of 16 November 2016 on delegation of the power to adopt fit and proper decisions and the assessment of fit and proper requirements (ECB/2016/42) and Decision (EU) 2017/936 of the European Central Bank of 23 May 2017 nominating heads of units to adopt delegated fit and proper decisions (ECB/2017/16).

30 Principle 3 of ECB (2017); this Guide was updated by ECB (2018b). On December 2021, the ECB published a new Guide to fit and proper assessments, ECB (2021). Furthermore, the ECB has expressed the request that the current lack of uniformity in the verification of the FAP requirements be overcome by updating the CRD IV: indeed, ‘while the guide will help to make such assessments in a more harmonised manner, discrepancies are set to remain on account of the application of national laws’, ECB (2018c), p 77; in the same sense, Brescia Morra et al. (2017), p 585.

31 As mentioned by the Guide itself: ‘This Guide covers fit and proper assessments of members of the management body, both in their management function (executive directors) and supervisory function (non-executive directors) of all institutions under the direct supervision of the ECB (significant institutions), whether credit institutions or (mixed) financial holding companies and in the case of licensing or qualifying holding procedures. On the basis of Article 6(4) of the SSM Regulation, responsibility for regular appointments in less significant institutions (i.e. outside the context of licensing or qualifying holdings) lies with the NCAs’, ECB (2021), p 6.

32 Principle 2, ECB (2017).

33 ECB (2021), p 12.

34 Ibid., p 14.

35 Calandra Buonaura (2019), p 30.

36 Associazione Bancaria Italiana (2017), p 6.

37 With reference, respectively, to banks based in Germany, Spain and the United States, see Hau and Thum (2009); Cuñat and Garicano (2010); Fernandes and Fich (2016).

38 For other suggestions, see Finesi (2015); Enriques and Zetzsche (2015), p 218 ff.

39 Smits (2020), p 10.

40 However, it should be noted that since November 2014, ‘12,567 individual assessments have been conducted’ and that ‘[s]ince its inception, the SSM has not issued negative decisions related to fitness and propriety. However, since the beginning of the SSM more than 50 applications were withdrawn or ended up in suspension or resignation of board members following interaction with the candidate or the bank’; basically, only 0.4% of the total applications were withdrawn, see Enria (2020), p 1 f.

41 See Bainbridge (2008).

42 Regarding technical skills, the ECB adopted a very general approach, see ECB (2021), p 9.

43 See EBA (2018), paras. 46, 51 and 53, respectively for the active involvement of independent members of the management body in special committees, and for the chairing of nomination and risk committees; and see the Joint ESMA and EBA (2017), para. 45, which states that ‘independent directors within the supervisory function of the management body help to ensure that the interests of all internal and external stakeholders are considered’. The different approach to formal independent board members across EU jurisdictions is clearly depicted by the competent authority’s comments in the confirmation of compliance with the EBA Guidelines, i.e., non-compliance by the German competent authority which states ‘as regards formal independence, the intended supervisory added value is not evident’.

44 With explicit reference to the implications of bail-in, Carriero (2017), p 642. More generally, in case law: ECtHR, 13 January 2009, *Todorova v Italy*, no. 33932/06; ECtHR, 10 April 2007, *Evans v United Kingdom*, no. 6339/05. In the sense that respect for fundamental rights constitutes a limit to the discretion of the authorities, Lehmann (2017), p 124 f.

45 For a general in-depth study of the Court of Justice’s scrutiny regarding compliance with the principle of proportionality, Wollenschläger (2014) and Sauter (2013); on the role played by the principle of proportionality in European Union law, see Tridimas (2006), p 136.

46 ECJ, 8 June 2010, *Vodafone Ltd and others v Secretary of State for Business, Enterprise and Regulatory Reform*, C-58/08, ECLI:EU:C:2010:321; ECJ, 7 July 2009, *S.P.C.M. SA, C.H. Erbslöh KG, Lake Chemicals and Minerals Ltd and Hercules Inc. v Secretary of State for the Environment, Food and Rural Affairs*, C‑558/07, ECLI:EU:C:2009:430; ECJ, 6 December 2005, *ABNA and others v Secretary of State for Health and Food Standards Agency*, C-453/03, C-11/04, C-12/04 and C-194/04, ECLI:EU:C:2005:741.

47 ECtHR, 16 June 2015, *Sargsyan v Azerbaijan*, no. 40167/06; ECtHR, 15 April 2014, *Stefanetti and others v Italy*, no. 21838/10, 21849/10, 21852/10, 21822/10, 21860/10, 21863/10, 21869/10 and 21870/10; ECtHR, 31 May 2011, *Maggio and others v Italy*, no. 46286/09, 52851/08, 53727/08, 54486/08 and 56001/08; ECtHR, 12 October 2004, *Kjartan Ásmundsson v Iceland*, no. 60669/00; ECtHR, 23 September 1982, *Sporrong & Lönnroth v Sweden*, no. 7151/75.

48 See ECtHR, 15 April 2014, *Stefanetti and others v Italy*; ECtHR, 31 May 2011, *Maggio and others v Italy*; ECtHR, 22 September 2005, *Goudswaard-Van der Lans v the Netherlands*, no. 75255/01.

49 ECB (2021), p 14.

50 Ibid., p 10.

51 Principle 4, ECB (2017), p 8. In the same sense, Joint ESMA and EBA (2017), pp 11 and 23.

52 On this point, Arrigoni (2020).

53 The European institutions themselves have this goal on their agenda, see Euro Summit (2018).

54 The classic debate on standards versus rules has already been mentioned above in n. 9.

55 Art. 10 of Law No 3864/2010 on the establishment of the Hellenic Financial Stability Fund, FEK A 119/21.07.2010 (HFSF).

56 The expression ‘competition in laxity’ means the circumstance whereby the competition at European level among banks involves the production and application of laws at national level aimed at stimulating the competitiveness of the own sector, while damaging its solidity and stability. On this topic observations are widespread: *ex multis*, see Moloney (2011) and Capolino (2016), p 58.

57 See Nouy (2018), stating that ‘we need to turn the rulebook for banks into a truly European one’.

58 ECB (2021), p 5.

59 On the other hand, there are problems as regards excessive harmonisation. More specifically, it does not allow for the specificities of each individual country to be taken into account (e.g., the role of independent directors varies according to national laws); furthermore, it requires identifying the solution to the problem of what is the best system to adopt; and finally it presents the risk of influencing the entire market through a single discipline.

60 In the sense that the bail-in tool is a ‘game changer’ able to move negotiations towards a more supranational political solution, reducing the costs of resolution in the euro area for the fiscally stronger countries, Quaglia and Spendzharova (2017), p 1122. Numerous in-depth analyses concern the subject of political interests in the process of integrating the single market in the financial sector; underlining the contrast between two different economy models, often identified as ‘market-making’ (or liberal market economy) and ‘market-shaping’ (or coordinated market economy), Quaglia (2010), p 1011, Moloney (2014), p 14 f. and Hooghe and Marks (1999), p 123; *contra*, in the sense that the two models of capitalism are not threatened by the path of integration of the single market (‘neither variety is likely to be in mortal peril’), Snell (2011), p 431.

61 See Costi (2019).

62 Binder (2019), p 36 ff. Additional references can be found in Busch et al. (2019), p 7 f.

63 Which is, in fact, relevant because among the 45 withdrawals of candidates for positions on the management body, 35% were due to the reputation criterion, see Enria (2020), p 4.

64 See Palm-Steyerberg and Busch (2019), p 199.

65 ECB (2021), p 14.

66 Significantly, the ECB (2021), p 14 ff, requires that there be elements that give rise to material doubt as to the reputation of the appointee (in fact, if the assessment raises material doubts, this should result in a negative decision: p 18). Our criteria can help to refine the supervisor’s assessment.

67 In this regard it should be noted that the by-laws and management board’s internal regulations of the supervised banks, particularly those with the most complex organisational and business structures, already delegate the decision-making on several matters to special committees, thus implicitly recognising the functions of their chairs.

68 It means that ‘they should be well informed about all the activities of their bank, actively seek out information and challenge the proposals of executive managers if necessary (‘nose in’). In this context, members of risk and audit committees should have close and regular contact with the heads of internal control functions. At the same time, non-executives must be careful to avoid becoming directly involved in the running of the bank and acting as a second executive team (‘fingers out’)’, ECB (2019c).

69 Indeed, ‘the Guidelines do not harmonise the point in time when assessments of the suitability of members of the management body should be made’, Joint ESMA and EBA (2021), p 14.

70 See Joint ESMA and EBA (2017), p 79 ff.

71 Ibid., p 80.

72 The costs are highlighted in Joint ESMA and EBA (2017), p 80.

73 Joint ESMA and EBA (2017), p 80.
